# Hospitalization among street-involved youth who use illicit drugs in Vancouver, Canada: a longitudinal analysis

**DOI:** 10.1186/s12954-018-0223-0

**Published:** 2018-03-20

**Authors:** Derek C. Chang, Launette Rieb, Ekaterina Nosova, Yang Liu, Thomas Kerr, Kora DeBeck

**Affiliations:** 1 British Columbia Centre on Substance Use, British Columbia Centre for Excellence in HIV/AIDS, St. Paul’s Hospital, 608-1081 Burrard Street, Vancouver, BC V6Z 1Y6 Canada; 2 Department of Family Practice, St. Paul’s Hospital, University of British Columbia, 608-1081 Burrard Street, Vancouver, BC V6Z 1Y6 Canada; 3 Department of Medicine, St. Paul’s Hospital, University of British Columbia, 608-1081 Burrard Street, Vancouver, BC V6Z 1Y6 Canada; 4 School of Public Policy, SFU Harbour Centre, Simon Fraser University, 515 West Hastings Street, Suite 3271, Vancouver, BC V6B 5K3 Canada

**Keywords:** Youth, Hospitalization, Mental illness, Drug overdose, Homeless, Cocaine

## Abstract

**Background:**

Street-involved youth who use illicit drugs are at high risk for health-related harms; however, the profile of youth at greatest risk of hospitalization has not been well described. We sought to characterize hospitalization among street-involved youth who use illicit drugs and identify the most frequent medical reasons for hospitalization among this population.

**Methods:**

From January 2005 to May 2016, data were collected from the At-Risk Youth Study (ARYS), a prospective cohort study of street-involved youth in Vancouver, Canada. Multivariable generalized estimating equation (GEE) was used to identify factors associated with hospitalization.

**Results:**

Among 1216 participants, 373 (30.7%) individuals reported hospitalization in the previous 6 months at some point during the study period. The top three reported medical reasons for hospital admission were the following: mental illness (37.77%), physical trauma (12.77%), and drug-related issues (12.59%). Factors significantly associated with hospitalization were the following: past diagnosis of a mental illness (adjusted odds ratio [AOR] = 1.85; 95% confidence interval [95% CI] 1.47–2.33), frequent cocaine use (AOR = 2.15; 95% CI 1.37–3.37), non-fatal overdose (AOR = 1.76; 95% CI 1.37–2.25), and homelessness (AOR = 1.40; 95% CI 1.16–1.68) (all *p* < 0.05).

**Conclusions:**

Findings suggest that mental illness is a key driver of hospitalization among our sample. Comprehensive approaches to mental health and substance use in addition to stable housing offer promising opportunities to decrease hospitalization among this vulnerable population.

## Background

Youth who are street-involved, defined as being homeless or using services for homeless youth, experience excess morbidity and mortality relative to the general population of adolescents and young adults [1, 2]. Many health concerns have been identified among this population, including sexually transmitted infections, mental illnesses, intentional and unintentional injuries, and substance use [3, 4]. Several health issues are directly related to substance use, including overdoses, infections, and psychological distresses [4]. The experience of homelessness and mental illnesses, such as conduct disorders, anxiety disorders, and mood disorders, which are prevalent among homeless youth, may also contribute to or exacerbate health issues among this population [5]. Despite multiple health vulnerabilities, numerous barriers to accessing care exist, including inadequate transportation, cost, fear of judgment, and lack of trust [6, 7]. Consequently, street-involved youth are often reluctant to engage with health services and frequently delay seeking help until their health problems deteriorate, which increases the risk of hospitalization [6, 8]. This tendency also contributes to the economic burden of delaying care among this population [4, 9].

Hospitalization among adult homeless populations has been well studied, while there is a paucity of similar research among homeless youth populations [10–12]. In the USA, it has been documented that acute medical conditions (primarily infections), mental illness, substance use, and injuries are the most common reasons for hospitalization among homeless youth [10]. To our best knowledge, no study has longitudinally examined hospital admissions among street-involved youth who use drugs in a Canadian setting with universal health care. In the context of growing concerns of homelessness and its related burdens, especially overdoses, among youth in North America [12], we sought to update the knowledge of the most common medical reasons for hospital admission and characterize hospitalization among street-involved youth who use drugs. We hope the findings can inform policy makers and healthcare workers to provide more effective interventions to prevent more serious health conditions requiring hospitalization among this vulnerable population and subsequently reduce the related economic burden.

## Methods

The At-Risk Youth Study (ARYS) began in 2005 and is an ongoing open prospective cohort study of street-involved youth in Vancouver, Canada. This study has been described in detail previously [13]. In brief, snowball sampling and street-based outreach as well as self-referral are used to recruit participants into the study. Persons between 14 and 26 years of age who have used illicit drugs other than or in addition to cannabis in the past 30 days and provide informed consent are eligible to participate. At baseline and semiannually thereafter, participants complete an interviewer-administered questionnaire. The questionnaire elicits sociodemographic data as well as information regarding participants’ substance use and other behavioral and socioeconomic data such as housing and engagement with health and social services. All participants receive a monetary stipend of $30 Canadian Dollars after each interview (in June 2016, the stipend amount was increased to $40). The University of British Columbia/Providence Health Care Research Ethics Board approved the study.

Data for this study was collected between January 2005 and May 2016. The primary outcome was self-reported hospitalization during the preceding 6 months. Specifically, participants were asked, “Have you been admitted to hospital in the last six months (yes vs. no)?” Participants who responded affirmatively were then asked to report the reason for hospitalization. These descriptive data were analyzed and grouped to identify the most common reasons for hospitalization among this sample.

To characterize hospitalization (which was self-reported and captured admissions to hospital), we considered a range of variables potentially associated with hospitalization. These were all measured within the preceding 6 months and included the following: any injection drug use, daily heroin use, daily non-medical prescription opioid use, daily crystal methamphetamine use, daily cocaine use, daily crack use, non-fatal overdoses, homelessness, living in the Downtown Eastside (DTES) neighborhood (Vancouver’s drug use epicenter), and incarceration (being in detention, prison, or jail). The following sociodemographic characteristics measured at baseline were also considered: age, gender, ethnicity, self-identified as LGBT (lesbian, gay, bisexual, and transgender), and self-reported diagnosis of a mental health illness. Covariates were selected based on review of the prior available literature [2, 3, 14].

Since analyses of factors potentially associated with hospitalization included serial measures for each subject, we used generalized estimating equations (GEE) for binary outcomes with logit link function for the analysis of correlated data. These methods determine factors associated with hospitalization throughout the greater than 11-year follow-up period and provide standard errors adjusted by multiple observations per person using an exchangeable correlation structure. Therefore, this analysis considered data from every participant follow-up visit. First, we used GEE bivariate analysis to determine factors associated with hospitalization. To adjust for potential confounding, all variables that were significant at *p* < 0.10 level in GEE bivariate analyses were considered in a full model. Quasi-likelihood under the Independence model Criterion (QIC) statistic with a backward model selection procedure was used to screen all possible combinations of candidate variables and identify the model with the best overall fit as indicated by the lowest QIC value. Analyses were performed using R version 3.2.4 (R Core Team (2016). R: A language and environment for statistical computing. R Foundation for Statistical Computing, Vienna, Austria). All *p* values were two-sided, and tests were considered significant at *p* < 0.05.

## Results

Overall, 1216 individuals completed follow-up visits including 380 (31%) female and 819 (67%) Caucasian youth. The median age of participants at baseline was 22 years (interquartile range [IQR] = 20–24). Participants contributed 4956 observations during the study period. The median number of follow-up visits was 3 (IQR = 1–5), and the median number of months between study follow-up was 6 (IQR = 6–8). Participants who did not return for a subsequent follow-up visit after baseline were significantly less likely to identify as LGBTQ and significantly more likely to be HCV positive (both *p* <0.05), though no other significant differences were observed between the two groups. Among our sample, 373 individuals (30.7%) reported being hospitalized at some point during the study period. Over the study period, these 373 participants contributed a total of 564 (11.4%) study observations that included a report of hospitalization. At baseline, 900 participants (74%) reported being homeless. At the most recent study visit during the study period, 111 of 272 participants (41%) reported being homeless (55% of the hospitalized group and 45% of those who reported no hospitalization during the study period).

Table 1 presents sociodemographic characteristics, drug use and socioeconomic factors at baseline, comparing those who did and did not report hospitalization during follow-up. Table 2 displays unadjusted and adjusted odds ratios for hospitalization and variables of interest. The adjusted multivariate model demonstrates that the youth who had a past diagnosis of a mental illness (AOR, 1.85; 95% CI, 1.47–2.33), used cocaine daily (AOR, 2.15; 95% CI, 1.37–3.37), experienced a non-fatal overdose (AOR, 1.76; 95% CI, 1.37–2.25), or were homeless (AOR, 1.40; 95% CI, 1.16–1.68) were significantly more likely to report recent hospitalization within the previous 6 months.

**Table 1:** Baseline characteristics (reported at time of study enrolment) of street-involved youth who report hospitalization during study follow-up: At Risk Youth Study (ARYS), Vancouver, British Columbia, 2005–2016 (*n* = 1216)

Characteristic	Hospitalized		
	Yes (%) (*n* = 151)	No (%) (*n* = 1065)	*p* value
Median age, years (IQR)	22 (20–23)	22 (20–24)	0.354
Female gender	54 (35.8)	326 (30.6)	0.201
Caucasian ethnicity	102 (67.5)	717 (67.3)	0.993
Identified as LGBT	120 (79.5)	872 (81.9)	0.301
Mental illness history	91 (60.3)	542 (50.9)	0.031*
Any injection drug use^‡^	54 (35.8)	348 (32.7)	0.460
Daily heroin use^‡^	24 (15.9)	112 (10.5)	0.046*
Daily prescription opioid use^‡^	6 (4.0)	36 (3.4)	0.694
Daily crystal meth use^‡^	25 (16.6)	137 (12.9)	0.223
Daily cocaine use^‡^	8 (5.3)	29 (2.7)	0.080
Daily crack use^‡^	29 (19.2)	157 (14.7)	0.166
Non-fatal overdose^‡^	31 (20.5)	139 (13.1)	0.015*
Homeless^‡^	122 (80.8)	778 (73.1)	0.052
Living in the DTES^‡^	48 (31.8)	303 (28.5)	0.397
Incarcerated^‡^	27 (17.9)	190 (17.8)	0.974

*p < 0.05

^‡^During the preceding 6 months

**Table 2:** Bivariable and multivariable GEE analyses of factors associated with hospitalization among street-involved youth: At-Risk Youth Study (ARYS), Vancouver, British Columbia, 2005–2016 (*n* = 1216)

Characteristic	Unadjusted OR (95% CI)	Adjusted OR (95% CI)^†^	*p* value
Age (per year older)	1.00 (0.97–1.02)		
Female gender	0.90 (0.72–1.13)		
Caucasian ethnicity	1.11 (0.88–1.40)		
Identified as LGBT	0.89 (0.68–1.18)		
Mental illness history	1.87 (1.50–2.35)	1.85 (1.47–2.33)	< 0.001*
Any injection drug use^‡^	1.29 (1.05–1.57)		
Daily heroin use^‡^	1.11 (0.87–1.41)		
Daily prescription opioid use^‡^	1.46 (0.88–2.42)		
Daily crystal meth use^‡^	1.13 (0.88–1.45)		
Daily cocaine use^‡^	2.36 (1.50–3.70)	2.15 (1.37–3.37)	0.001*
Daily crack use^‡^	1.13 (0.84–1.50)		
Non-fatal overdose^‡^	1.98 (1.55–2.51)	1.76 (1.37–2.25)	< 0.001*
Homeless^‡^	1.41 (1.18–1.68)	1.40 (1.16–1.68)	< 0.001*
Living in the DTES^‡^	1.11 (0.90–1.36)		
Incarceration^‡^	1.11 (0.87–1.43)		

*p < 0.05

^†^Variables significant at *p* < 0.10 in bivariate models were eligible for possible inclusion in the multivariable model; variables included in the final multivariable model were identified using a backward selection approach to minimize the Quasi-likelihood under the Independent model Criterion (QIC)

^‡^During the preceding 6 months

Table 3 displays the top medical reasons for hospitalization. Mental illness (37.77%) was the most common medical condition followed by physical trauma (12.77%) and drug-related conditions (12.59%).

**Table 3:** Top five medical reasons for hospitalization among street-involved youth: At-Risk Youth Study (ARYS), Vancouver, British Columbia, 2005–2016 (*n*=373 participants who contributed 564 study observations)

Medical condition	*N* (%)
Mental illness	213 (37.77)
Physical trauma	72 (12.77)
Drug related	71 (12.59)
Infection related	48 (8.51)
Pregnancy related	35 (6.21)

Based on total number of reports of hospitalization, not number of participants

## Discussion

Based on this prospective cohort of street-involved youth who use drugs, homelessness, past diagnosis of a mental illness, frequent cocaine use, and non-fatal overdose were significantly associated with hospitalization. Among the medical reasons for hospital admission, mental illnesses, physical trauma, and drug-related conditions were the most common reasons.

Our finding that homelessness was significantly associated with youth hospitalization is consistent with previous literature indicating that homelessness and unstable housing not only increase hospital use but also increase the length of hospital stay [9, 14]. Conversely, the longer their stay in the hospital, the more likely the youth could lose their housing. Our results build on a significant body of research highlighting the essential role of stable housing in supporting the health and well-being of vulnerable populations. Evidence also suggests that the relationship between mental illness and homelessness can be multidirectional. Homelessness is known to directly undermine mental health [15], and mental illness can directly contribute to becoming homeless [15]. Regardless of the direction of the relationship, a multisite randomized controlled study demonstrates that a “housing first” approach (combined with assertive community treatment or intensive case management) improves housing stability among homeless youth with mental illnesses [16].

Our study also shows that a history of mental illness was significantly associated with hospitalization among street-involved youth, and mental illness was the number one reason for hospital admission. While this correlation has been established among adult homeless populations, it has been less clear among homeless youth [17]. Previous literature demonstrates that the prevalence of psychiatric disorders is high (88%) among homeless youth, but only 31% had accessed any form of mental health services, including in community clinics, emergency rooms, or addiction treatment [18]. Our findings therefore point to the importance of improving access to mental health services in the community among street-involved youth.

Substance use-related issues, specifically frequent cocaine use and non-fatal overdoses, were also associated with youth hospitalization among our sample. Evidence has shown that illicit substance use is associated with increased risk of hospitalization [19]. Our study contributes to the knowledge that cocaine in particular is associated with increased risk of hospitalization. Previous literature also highlights other risks associated with stimulant use among homeless youth, particularly risky sexual behaviors and incarceration [20]. Given these harms, it is particularly concerning that evidence-based treatment options for stimulant use disorder are limited; innovation in this area is needed.

Moreover, non-fatal overdose was independently associated with hospitalization among street-involved youth. Drug-related overdoses continue to increase among young adults and adolescents who use drugs, and opioid overdoses have risen at an alarming rate in recent years in Vancouver [21–23]. Extensive morbidity is also associated with non-fatal overdoses, including physical injury, falling, or personal assaults [24]. Overdose prevention and harm reduction programs, such as peer-based education and naloxone training, as well as expanding treatment options, can be expected to help decrease youth hospitalization [25, 26].

In our study among street-involved youth engaged in illicit substance use, over 50% reported a history of mental illness at baseline indicating that the prevalence of dual diagnosis among our sample is high. This is consistent with the literature that reports the prevalence of dual diagnosis between 35 and 76% among homeless youth [4]. A recent study also found that precariously housed youth were 48% more likely to be diagnosed with dual diagnosis [27]. Given the prevalence of youth with dual diagnosis, it is essential to integrate interventions such as systematic screening for dual diagnosis and building mental health and addiction training into youth-serving organizations [27]. Early diagnosis can also increase the likelihood that youth will access care before the condition progresses further and requires hospitalization.

When trying to access healthcare, youth are known to face several barriers including financial (lack of free transportation or health insurance), structure (unable to obtain birth certificate or ID), and personal (lack of knowledge or fear of judgment from healthcare professionals) [4, 6, 7]. Thus, it is critical to ensure that youth do not face restrictive barriers when trying to access services, so youth can access early interventions to prevent eventual hospitalizations.

There are several limitations to this study. First, the ARYS cohort is not a random sample. Therefore, study findings may not generalize to other populations. Second, the results are based on self-reported data, which may be affected by recall bias and socially desirable responding. Also, they may not accurately reflect the medical reasons for hospitalization. Third, as with all observation studies, the independent associations that we found could have been influenced by other confounding variables.

## Conclusions

Our study suggests that mental illnesses were a key driver of hospitalization among street-involved youth. Frequent cocaine use, non-fatal drug overdose, and homelessness were also significantly associated with hospitalization. Based on these findings, promising opportunities to prevent hospitalization may include ensuring stable housing in line with the “housing first” approach, increasing access to youth-friendly mental health and addiction treatment services, providing overdose prevention education and harm reduction measures, and improving early identification of dual diagnosis and access to care.
